# A review on perovskite oxides and their composites as electrode materials for supercapacitors

**DOI:** 10.1039/d5ra01950h

**Published:** 2025-05-21

**Authors:** Mugil Neelakandan, Preethi Dhandapani, Senthilkumar Ramasamy, Ramesh Duraisamy, Seung Jun Lee, Subramania Angaiah

**Affiliations:** a Electro-Materials Research Laboratory, Centre for Nanoscience and Technology, Pondicherry University Pondicherry-605 014 India subramania@gmail.com; b Department of Physics, National Central University Taoyuan 32054 Taiwan; c Center of Excellence in Advanced Materials and Green Technologies, Amrita School of Engineering, Amrita Vishwa Vidyapeetham University Coimbatore-641112 India; d Dept of Chemistry, College of Natural & Computational Sciences, Arba Minch University P.O. Box 21 Arba Minch Ethiopia ramesh.duraisamy@amu.edu.et; e Dept of IT-Energy Convergence (BK21 FOUR), Korea National University of Transportation Chungju-27469 Republic of Korea sjlee@ut.ac.kr

## Abstract

In energy storage applications, supercapacitors serve as an alternative to electrochemical batteries due to their large power density and exceptionally long cycle life. Redox-active supercapacitors are favoured for their durability and power density arising from the carbon-dominated field. However, their commercialization is questioned due to their slow reaction kinetics and low energy density limitations. Electrode materials with superior electrochemical behaviour must be developed to overcome these obstacles. The oxygen anion-intercalation mechanism leads to an interest in perovskite oxide materials with intrinsic oxygen vacancies and flexible structural characteristics. The primary objective of this review is to present an overview of the fundamental characteristics of perovskite oxides, their charge storage mechanism, and the key factors governing the electrochemical behaviour of the active material. This review was also compiled by reviewing previous research on perovskite materials for supercapacitors. This study examines the anion-intercalation mechanism and the variables affecting the electrochemical performance of electrodes. Furthermore, this review addresses the challenges and significance of previous research. Moreover, it presents the design guidelines for perovskite materials for supercapacitors, which appear beneficial for future studies on these materials.

## Abstract 2

Perovskite oxides and their composites enable high capacitance, redox activity, and cycling stability, making them ideal for advanced supercapacitor electrodes.

## Graphical abstract

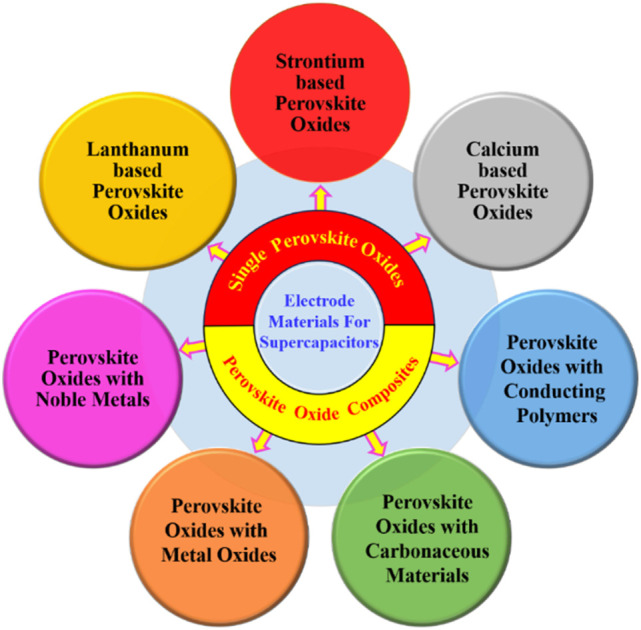

## 1. Introduction

In the field of energy conversion and storage, supercapacitors (SC), Li-ion batteries, and fuel cells are gaining more attention. To close the gap between conventional capacitors and rechargeable batteries, SCs are viewed as a new kind of energy storage device because of their high-power density, ultrafast charge–discharge rate, low internal resistance, long cycling life, and wide operating temperature (from −40 to 70 °C).^1–5^ SCs generally operate through two distinct mechanisms: Electric Double-Layer Capacitance (EDLC) and pseudo capacitance. The fundamental mechanism in EDLCs is electrostatic adsorption, which results in the formation of charges at the electrode–electrolyte interface.^6,7^ Conversely, pseudocapacitors have charge storage through the faradaic reaction between electroactive species, which helps in achieving better charge density. The charge storage capacity of pseudocapacitors, which relies on redox reactions, surpasses that of EDLC by 10–100 times.^8,9^ This significant enhancement is attributed to the rapid faradaic process employed in pseudocapacitance for charge storage, as opposed to the electrostatic adsorption mechanism in EDLC.^10,11^ Although Transition Metal Oxides (TMOs), including MnO_2_ and Co_3_O_4_, are considered cost-effective and promising electrodes for SC, their widespread adoption in SC applications is significantly hindered by their poor energy density and limited cycling lifespan.^12–14^ During redox reactions in perovskite oxides (PO) within the electrolyte, the oxygen vacancies associated with the oxygen ion intercalation mechanism explain the oxygen ion insertion and extraction process.^15^ As oxygen vacancies carry charges, it is well known that controlling their concentration is one of the most efficient and simplest paths for increasing the capacitance of the materials used for oxygen ion intercalation.^16^ A-Site and B-site doping techniques in ABO_3_ have been demonstrated to modify intrinsic physicochemical properties, including electrical conductivity, catalytic activity, ferromagnetism, and oxygen vacancies.^17^ Moreover, constructing lattice distortion can produce abundant oxygen vacancies. On the other hand, oxygen ion's insertion and extraction capabilities are influenced by the variable valence of B-site ions which is observed in the lattice of perovskite-type oxides.^18^ As a result, hetero ion doping can alter the perovskite's lattice structure and improve its electrochemical characteristics.^19^ The primary PO for supercapacitors includes single perovskite oxide (ABO_3_), double perovskite oxide (AA′BB′O_6−*δ*_), triple perovskite oxide (AA′A′′BB′B′′O_9−*δ*_), Ruddlesden–Popper (RP, A_2_BO_4−*δ*_), and other derived perovskite-type oxides.^20–22^ Single perovskite oxides have been extensively studied in lanthanide-based (La-based) perovskites, including LaMnO_3_, LaFeO_3_, LaCoO_3_, and LaNiO_3_, due to their high voltage window and excellent stability. LaNiO_3_ demonstrated the highest specific capacitance (*C*_sp_) of 719 F g^−1^ in neutral electrolyte.^23,24^ However, leaching cations in the aqueous electrolyte can damage the crystal structure of a single perovskite oxide.^25^ The electrochemical stability of SC is enhanced by the more stable structure of double and triple perovskite oxides, which feature a mixed arrangement of distinctions.^26,27^ Moreover, they exhibit wider voltage windows, which increases the energy density of SC according to the energy density calculation formula, *E* = ½*CV*^2^, where *C* and *V* represent the working electrode's specific capacitance (*C*_sp_) and potential window, respectively, and also highlight the SC's application potential.^28,29^ Meanwhile, the power density is calculated by the formula *P* = (*E* × 3600)/Δ*t* where *E* and Δ*t* represents energy density and discharge time. In particular, under various reduction conditions, the rapidly scattered lattice elements of a simple double perovskite oxide (AA′B_2_O_6_) can be transformed into an ordered structure with a uniform distribution of A-site cations. Faster oxygen kinetics are promoted by the internally ordered structure of double perovskite oxides compared to the disordered structure in solid oxide fuel cells.^30,31^ For SC, the ordered structure facilitates the concentration of oxygen vacancies. Sr_2_CoMoO_6_ and PrBaMn_2_O_6_, two prominent double perovskite oxides, have been studied as electrode materials.^32–35^ Sr_2_CoMoO_6_ demonstrates a B-site-ordered rock-salt structure, whereas PrBaMn_2_O_6_ exhibits an A-site-ordered structure. According to the description, the internally ordered structure can offer a more convenient electron transmission path and more oxygen vacancies than the disordered counterpart. Liu *et al.* noted an ultrahigh *C*_sp_ of 1571 F g^−1^ for Pr_0.5_Ba_0.5_Mn_1.7_Co_0.3_O_6−*δ*_ with Co_3_O_4_ on the double perovskite surface.^36^ It utilized the decay of specific transition metal ions in double perovskite oxide within an H_2_ atmosphere.^37^ The use of triple and double POs is expanding quickly, and more practical methods of enhancing their functionality in SC have been created.^36^ Moreover, as of the rapid development, a current conclusion is necessary to systematically describe the most recent development direction in SC with a perovskite oxide electrode in recent years. It will serve as both a rapid reference and an inspiration for creative approaches for future research. This paper compiles the modification guidelines for PO, including morphological design, doping strategy, and composite formation with other materials.^33^ The phase transition transformation process under a reduced environment and the modifications induced by the segregation of B-site metal ions are explicitly discussed for double perovskite oxide.

## 2. Types of perovskites

### 2.1 Single perovskite oxides

**Fig. 1:**
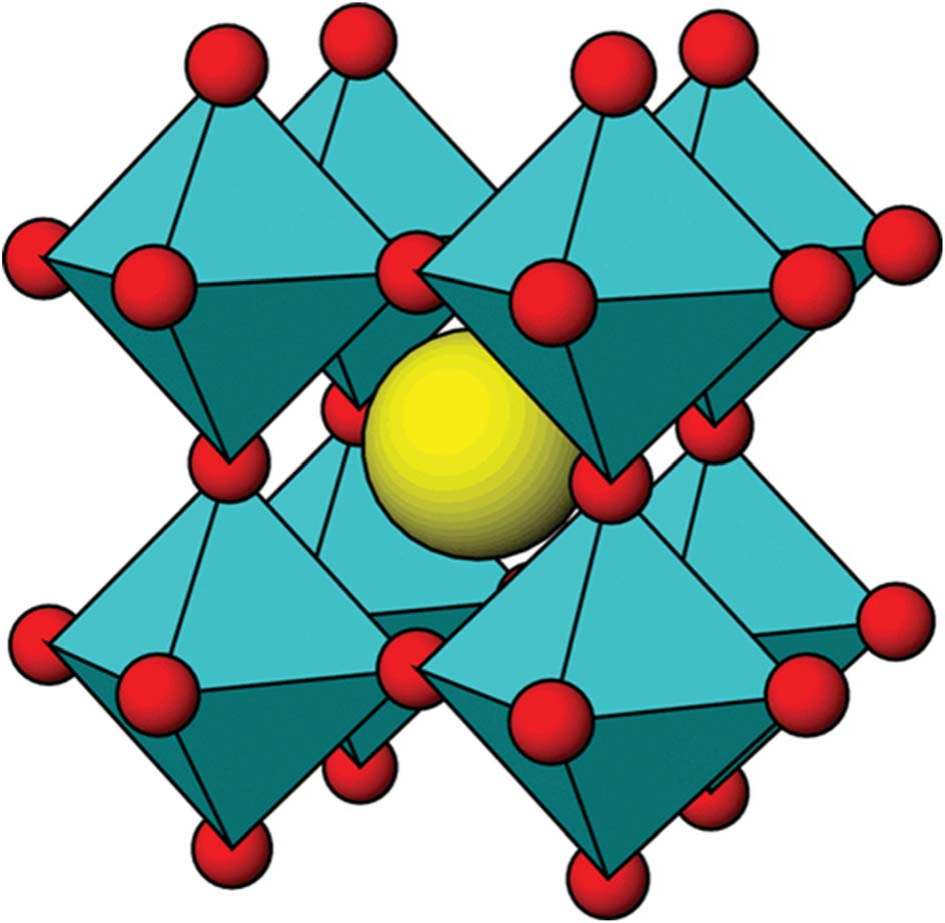
Ideal cubic perovskite structure for ABO_3_ (BO_6_ units – cyan; A atoms – yellow; O atoms – red).^38^ This figure has been reproduced from ref. 38 with permission from *Frontiers in Chemistry*, copyright 2025.

Perovskite materials have served as a substance for several decades. A perovskite structure embodies an ideal configuration characterized by an A-cubic crystal and ABO_3_ stoichiometry lattice, which belongs to the space group *Pm*3*m* (Fig. 1). The components of this structure consist of a three-dimensional framework created by corner-sharing BO_6_ octahedra. Perovskite oxide with the formula ABO_3_ has a distorted structure, oxygen vacancy concentration, and high tap density, where A represents the alkaline rare earth, and B represents the transition rare earth elements.^38^ Element A possesses a larger ionic radius than element B, and other metal elements can be substituted for the ions at both the A and B sites. PO doped with varying physicochemical characteristics, such as polarity, specific surface area, and electrical conductivity, enable the exploitation of this property.^38–40^ As a result, PO holds enormous promise for use in solar cells, superconductors, catalysts, and sensor materials.

**Fig. 2:**
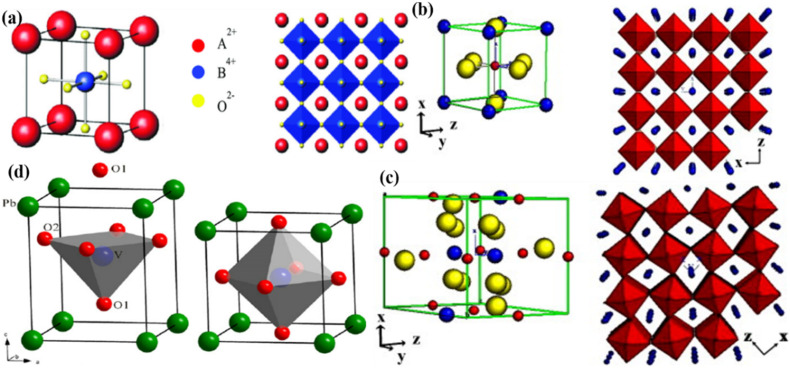
Different structures of perovskite material (a) cubic, (b) monoclinic, (c) orthorhombic and (d) tetragonal.^41,42^ This figure has been reproduced from ref. 41 and 42 with permission from Elseiver, copyright 2025.

The oxygen atom is present at the centre of each of the 12 sides, the centre of the cube's body is A-cations, and each of the eight corners is called B-cations while examining the structure of PO. Considering the A site at the cubic corner position (0, 0, 0), the face-centred position of the oxygen atom will be located at the cubic lattice (½, ½, 0), and the B cation occupies the body-centred position (½, ½, ½), while the A cation is visible with 12-coordination to the oxygen anion (Fig. 2). Therefore, the B–O bond distance is *a*/2, and the A–O bond distance is *a*/√2, where *a* is the cubic unit cell parameter.^41,42^ The radii of the A-site and B-site ions must satisfy the formula *t* = (*r*_A_ + *r*_*x*_)/√2(*r*_B_ + *r*_*x*_), which results in the formation of the required perovskite structure. The tolerance of the cubic system is *t* = 1, which represents the optimal value for the perovskite structure. Adjusting the octahedral factor *μ*-value (*μ* = *r*_B_/*r*_*x*_) between 0.44 and 0.90 and the *t*-value between 0.89 and 1.0 aids in forming the ideal cube-shaped perovskite structure and regulates the stability of the octahedra. Moreover, A–O and B–O bond lengths will vary when a significant portion of the atoms in the A and B sites are changed for other elements. Therefore, a popular technique for enhancing the properties of perovskites is to dope with numerous cations of different ionic radii and valence to substitute the A and B site cations fractionally. The current value of *μ* = 0.59 can form the BX_6_ structure, which enhances the stability of the perovskite. When tilting the crystal structure of perovskite ABO_3_, octahedra are formed, leading to the development of crystal phases such as triclinic, monoclinic, orthogonal, tetragonal, and rhombohedral (Fig. 2).^42–45^

### 2.2 Double perovskite oxides

**Fig. 3:**
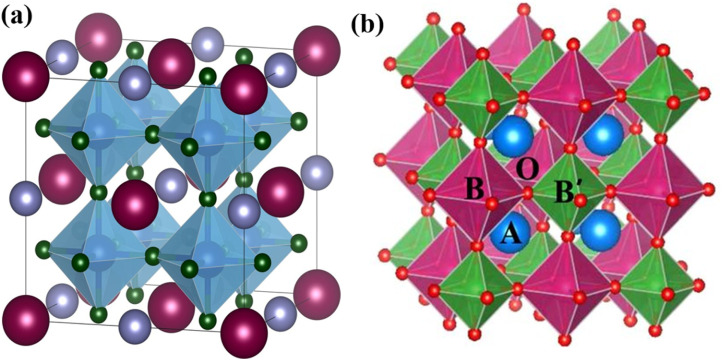
Schematic illustration of (a) A′A′′BO_6_ (double A site), (b) AB′B′′O_6_ (double B site).^46,47^ This figure has been reproduced from ref. 46 and 47 with permission from RSC, copyright 2025.

Perovskite oxides (ABO_3_) can result in the formation of double perovskite oxides when half of the B site cation is replaced by one cation.^38,42,45^ Meanwhile, the cations of the A and B sites are positioned in the resulting structures, such as A′A′′BO_6_ (double A site) and AB′B′′O_6_ (double B site) (Fig. 3).^48^ The expansion of a basic perovskite oxide unit results in the formation of double perovskite oxides, where the A and A′ cations are enclosed within cub-octahedrons, while the B and B′ cations occupy the central positions of octahedrons in the double perovskite oxide structure.^49,50^ The appropriately ordered B′ and B′′ cations are necessary for the double perovskite A_2_B′B′′O_6_ crystal structure.^46,47^ There are three primary types: layered B-cation sublattice structures, rock salt, and columns. Bochu *et al.* initially identified these materials, where an additional transition metal (the A′ site) occupies 75% of the A site.^51^ This can lead to the emergence of two possible structures: an A site-ordered quadruple perovskite 
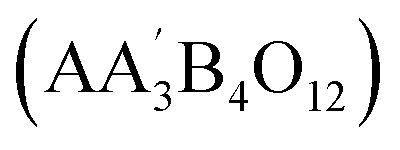
 or a 1 : 3-type A site cation arrangement 
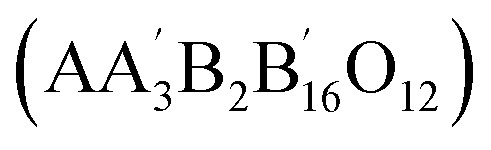
. High-pressure techniques are generally necessary to synthesize these quadruple perovskites, ensuring the cation is accurately positioned in the A′ site under square planar conditions.^51,52^ Alternatively, the B site is adaptable and can accommodate cation variability. One of the distinctive structural and electronic properties of these structured quadruple perovskites is A′–B intersite charge transfer. The A and B sites promote ferroelectricity, while the A′–B–B′ spin interactions are strengthened, leading to elevated spin ordering temperatures.^53–55^ Kumar *et al.* investigated the electrochemical performance of R_2_MMnO_6_ perovskite oxides (R = La, Gd; M = Zn, Cu, Ni). The prepared electrode namely La_2_ZnMnO_6_, La_2_CuMnO_6_, and Gd_2_NiMnO_6_ exhibited specific capacitances of 718.6 F g^−1^, 205.5 F g^−1^, and 400.46 F g^−1^, respectively, at varying current densities.^56,57^ Further, to enhance supercapacitor characteristics, improving specific surface area and increasing charge transfer at the nanoscale are found to be effective measures.^58,59^ Meng *et al.* synthesized a hollow spherical porous structure of La_2_CoMnO_6_ through template impregnation (HS-LCMO).^60^ In comparison, La_2_CoMnO_6_ was synthesized using the sol–gel method (SG-LCMO). The specific surface area of HS-LCMO and SG-LCMO was found to be 22.14 and 10.36 m^2^ g^−1^, respectively.^60^

### 2.3 Triple perovskite oxides

**Fig. 4:**
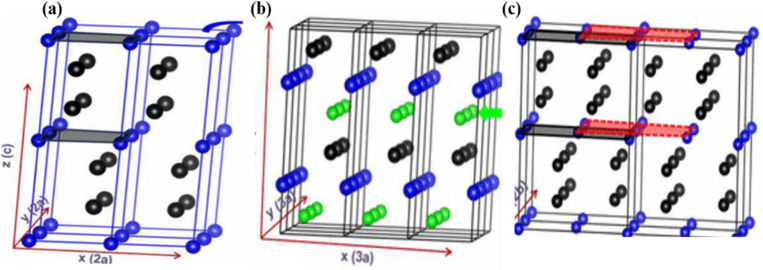
Schematic representation of (a) 6H-A and (b) 6H–B and (c) orthorhombic crystal systems.^61,62^ This figure has been reproduced from ref. 61 and 62 with permission from RSC, copyright 2025.

More precise configurations, such as triple POs 
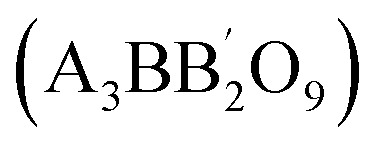
 and the perovskite family, offer a straightforward method for exploring structure–property relationships due to their ability to adopt various distinct stacking sequences. They can also accommodate a variety of metal cations, including those with unpaired electrons, in unique crystallographic positions with flexible bond angles.^49^ Their general formula is typically 
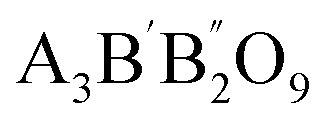
 or A_3_B_3_O_9_, where the specific arrangement of cations is crucial in determining the material's overall properties. The A-site cations (*e.g.*, La, Sr, Ba, Ca) typically occupy a cubic or distorted cubic lattice, providing structural stability. In contrast, two transition metal (B-site) cations occupy distinct positions within the octahedral network. The crystal structure is significant for basic and applied research in any chemical system, including triple perovskites.^43^ The triple perovskite oxide can be divided into hexagonal and non-hexagonal systems depending on the material's structural distortion. Most systems belong to hexagons, while others belong to non-hexagonal crystal structures like monoclinic and orthorhombic. The hexagonal triple perovskite may contain a 6H hexagonal structure with unique 3-fold or 6-fold rotational symmetry, which undergoes crystallization belonging to *P*6_3_/*mmc* and *P*6_3_*mc* space groups, respectively. These crystal systems are named 6H–A and 6H–B triple perovskite system.^61^Fig. 4 shows the schematic representation of (a) 6H–A and (b) 6H–B crystal systems. In non-hexagonal triple perovskite systems, the 6H–A crystal structure transforms to other structures like monoclinic or orthorhombic due to structural distortion, as shown in Fig. 4c. This primarily depends on the amplitude of distortion caused by the cationic size mismatches in the system and accompanied by the modulation of face-sharing octahedra-polymers.^61,62^

**Fig. 5:**
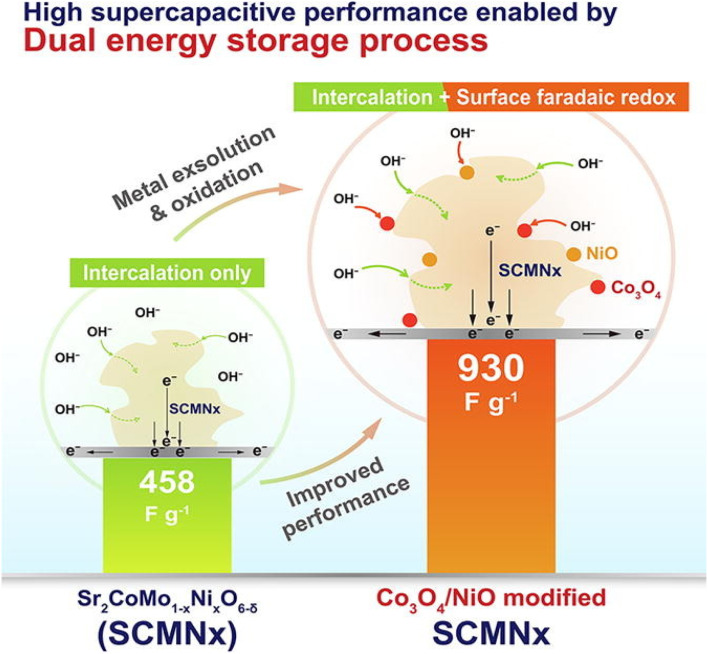
Charge storage mechanism of Sr_2_CoMo_1−*x*_Ni_*x*_O_6−*δ*_.^65^ This figure has been reproduced from ref. 65 with permission from Elseiver, copyright 2025.

The specific capacitance of PO has been significantly enhanced through the various modification techniques, leading to an expansion in the energy density of these materials. Among the key innovations is extending the voltage window, which directly contributes to improving the energy density of SC. The unique crystal structure of PO allows for incorporating different metal components, making them highly suitable for improving the voltage range in SC. A notable example is the triple perovskite oxide Sr_3_CoFeMoO_9−*δ*_, proposed by Qiao, demonstrating distinct redox potentials for its metal components—Co, Fe, and Mo. The specific arrangement of these metals, influenced by the Fe elements against inductive effects, leads to a long voltage window of 1.4 V for Sr_3_CoFeMoO_9−*δ*_ (SCFM). Furthermore, the voltage profile from the GDC curve demonstrated exceptional stability at different current densities, indicating consistent performance under varying conditions. The material exhibited excellent capacitance retention, with 74.6% of the *C*_sp_ preserved at a scanning speed of 10 mV s^−1^. These characteristics highlight the potential of PO, particularly SCFM, as advanced materials for next-generation supercapacitors with enhanced stability and energy density. The symmetrical supercapacitor SCFM‖SCFM exhibited a power density of 1412.9 W kg^−1^ and an energy density of 58.5 W h kg^−1^ within a potential window of 1.4 V.^63^ The supercapacitors fabricated with this electrode material demonstrated an *C*_sp_ of 685 F g^−1^ at a 2.0 A g^−1^ current density. The partial substitution of Mn^+^ at the B-site was pivotal in enhancing the oxidation state of Fe cations and promoting the mobility of oxygen ions through oxygen vacancy sites. Moreover, the electrochemical stability of LBFM-0.2 was rigorously evaluated by subjecting it to extended charge–discharge cycles, showcasing its potential for practical applications. After 3000 charge–discharge cycles, the supercapacitor retained approximately 94% of its initial capacity.^64^ Yin Qiao synthesized SrFe_1−*x*_Zr_*x*_O_3−*δ*_ by using a solid-state synthesis method. Zr substitution enhances the structural stability in comparison with the pure SrFeO_3−*δ*_. The fabricated electrode demonstrates a suitable *C*_sp_ of 163.92 F g^−1^ and excellent cycling stability.^63^ Liu *et al.* synthesized Sr_2_CoMo_1−*x*_Ni_*x*_O_6−*δ*_ using the sol–gel method. The prepared electrode enables two distinct forms of energy storage, including faradaic surface redox pseudocapacitance and oxygen anion-intercalation pseudocapacitance, as shown in Fig. 5. The electrode exhibits enhanced electrochemical performance, demonstrating a *C*_sp_ of 930 F g^−1^.^65^ Furthermore, Zhu *et al.* synthesized SrCo_0.9_Nb_0.1_O_3−*δ*_ (SCN) using the ball milling method, which exhibits stability at 95.7% of its original capacity after 3000 cycles with a high energy density of 37.6 W h kg^−1^.^64,66^ Hence, double perovskite oxide clarifies its well-ordered structure and specific surface modification engineering, whereas triple perovskite oxide offers the advantages of a diverse metal element arrangement.

## 3. Charge storage mechanism in perovskite oxides

**Fig. 6:**
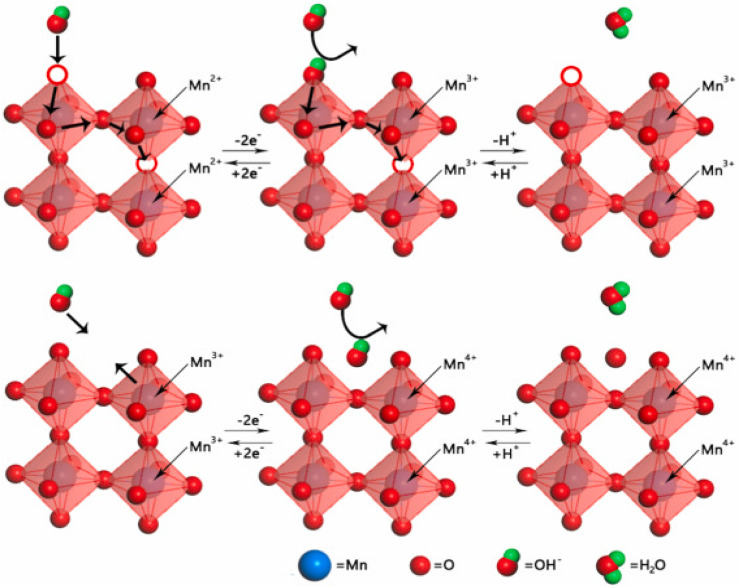
Schematic illustration of oxygen intercalation mechanism in PO.^70^ This figure has been reproduced from ref. 70 with permission from Elseiver, copyright 2025.

Due to their simple cost, large skeletal structure, high charge density, and basic properties like oxygen vacancies and chemical tunability, PO have attracted much interest.^67,68^ These materials have been widely used as useful substances in energy-related applications for several years. Meffold efforts towards the study of the oxygen anion intercalation process in nanostructured lanthanum-based PO was an important historical moment.^69^ The method follows like this: first, oxygen diffuses as OH^−^ ions from the electrolyte. Within the crystal lattice, these OH^−^ ions increase in the edges of the octahedral structures and fill the oxygen vacancies. Water is produced due to the oxidation of two Mn^2+^ ions to Mn^3+^ simultaneously. Intercalation of excess oxygen occurs at the material's surface in the next reaction phase. As shown in Fig. 6, this involves the oxidation of two Mn^3+^ ions to Mn^4+^ and the release of manganese to the surface.^70^ As mentioned earlier, most PO are essential for charge storage. Other metal oxides that rely on positive ions such as Li^+^ or Na^+^ for charge transfer, including TiO_2_–B, T–Nb_2_O_5_, and α-MoO_3_, demonstrated similar intercalation behaviour. On the other hand, O^2−^ ions in PO are very useful and can store multiple times as much charge per cycle as Li^+^ intercalation.^71,72^ PO are also considered upcoming materials for supercapacitors due to this characteristic. The idea is that O^2−^ ions have greater intercalation pseudocapacitance because they can carry two negative charges per unit.

## 4. Synthesis methods of perovskite oxides

### 4.1 Microwave synthesis method

**Fig. 7:**
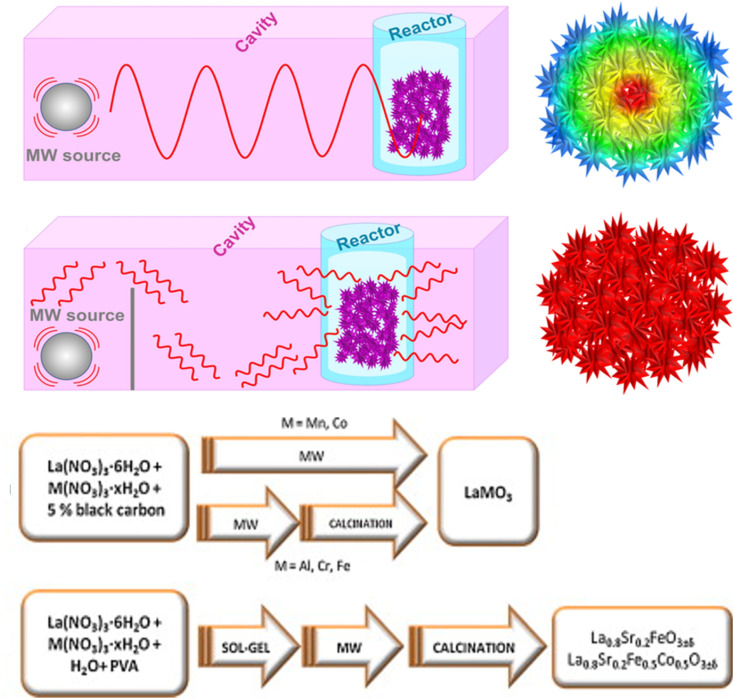
Schematic representation of the microwave synthesis method.^74,75^ This figure has been reproduced from ref. 74 and 75 with permission from RSC, copyright 2025.

There are several benefits of using vapour-phase synthesis for introducing and managing nanostructures. Electromagnetic waves with high frequency and wavelengths ranging from 1 mm to 1 m, specifically microwaves operating within the frequency range of 0.3 to 300 GHz, are utilized. They are frequently employed for both cooling and heating purposes. The microwave approach uses heating mechanisms, including ionic conduction and depolarization, which heat molecules or ions through collisions and friction. Chemical reactions are efficiently enhanced by this method.^73^Fig. 7 illustrates the schematic representation of the microwave synthesis method. Chen *et al.* used vapor-phase synthesis at high temperatures to synthesis CsSnI_3_, which displayed a low surface combination rate and a long carrier diffusion length (∼1 μm). The CsSnX_3_ precursor powder was heated to 280 °C and then produced to a mica substrate ([KAl_2_(Si_3_Al)O_10_(OH)_2_]) by flowing argon gas to enable the controlled growth of nanowires. Bulk synthesis, melting, and colloidal processes are standard synthesis techniques for PTO.^76^

Due to perovskite's outstanding characteristics that have made it easy to modify, several manufacturing methods have been developed, with the solvothermal approach being one of the most widely used. To create Ru-based perovskite/graphene nanocomposites, Hassan *et al.* used the redox reactions that were started by heating using thermal and microwave assistance. Both techniques utilized direct redox interactions between GO and Ru precursors.^74–76^ According to structural research and material characterizations, the microwave-assisted method developed more well-ordered nanoparticles, while thermal heating yielded better reduction efficiency.^77^ Kostyukhin *et al.* utilized microwave heating to synthesize lanthanum orthoferrite (LaFeO_3_) perovskites to address the issue of extended synthesis durations.^76^ This microwave method accelerates LaFeO_3_ particle growth and crystallization through surface polarization and localized overheating. This method provided a more efficient method for material creation than hydrothermal synthesis, decreasing the synthesis time by about 10 hours.^76^

### 4.2 Sol–gel method

**Fig. 8:**
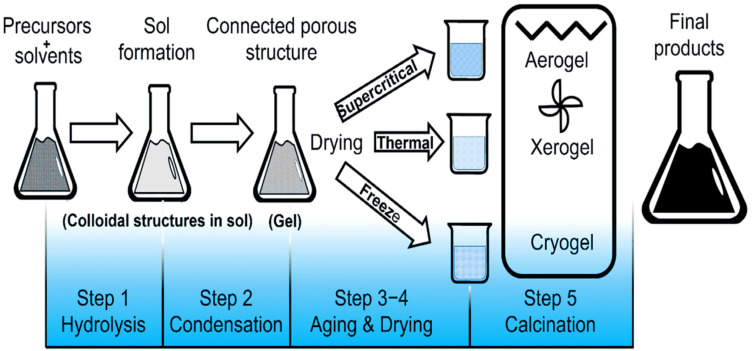
Schematic representation of POs by the sol–gel method.^80^ This figure has been reproduced from ref. 80 with permission from Elseiver, copyright 2025.

The sol–gel technique allows for precise control over composite particle's uniformity and particle size. However, it typically requires multiple complex agents, extended processing times, and stringent parameters such as specific temperatures and pH.^78^ Controlling the structure of oxides produced by the sol–gel technique is challenging, and the intrinsic instability of the structure restricts its application. Despite being highly homogenous and not requiring high processing temperatures, nanomaterials are frequently created in polycrystalline form.^79^Fig. 8 illustrates the schematic representation of PO using the sol–gel method.^80^ Kharangarh *et al.* used the sol–gel and hydrothermal methods in a process that involved two steps to create a high-conductivity electrode material. Initially, the SrCo_0_._9_Mo_0_._1_O_3−*δ*_ (SCM) perovskite had been generated by doping molybdenum into cobaltite using the sol–gel technique. Graphite was finally added to the SCM using the hydrothermal process. Due to increased oxygen vacancies, SCM showed better cycle life and specific 440 F g^−1^.^81^ Hu *et al.* synthesized CsPbX_4_ NCs in a covalent solvent to create advanced CsPbX_3_ NCs using a water-assisted transformation method. The surface of perovskite NCs at the hexane/water interface was then functionalized through an effective sol–gel modification approach. With this method, monodisperse CsPbX_3_/SiO_2_ and CsPbBr_3_/Ta_2_O_5_ were effectively produced. At the hexane/water contact, it is notable that change and oxide attachment took place at the same time.^82^ Zhang *et al.* successfully produced LaFeO_3_ perovskite nanoparticles using a sol–gel method by utilizing the porosity properties of MOF templates and advanced methods. The new technique performed exceptionally well, offering more relaxed preparation conditions, easier preparation, and flexible processing intervals. The procedure started with systematically mixing La salt, Fe salt, and the organic ligand H_3_BTC in an ethanolic solution to create the first product (MOG-La-Fe). After MOG-La-Fe was pyrolyzed on an appropriate substrate, mesoporous LaFeO_3_ perovskite nanoparticles were produced. At a power density of 900 W kg^−1^, these materials demonstrated outstanding energy efficiency of 34 W h kg^−1^.^83,84^ Tomar *et al.* synthesized SrTiO_3_ perovskite oxide nanofibers. The synthesis of SrTiO_3_ nanostructures was carried out by sol–gel method, then heating at various temperatures. Consequently, the generated electrode exhibits improved electrochemical characteristics, including increased *C*_Sp_, high-rate capability 208.47 F g^−1^, better cyclic stability at 1500 cycles and longer cycle life.^85^ Tomar *et al.* synthesized SrTiO_3_ using the sol–gel method for PO with a cubic structure. Consequently, the SrTiO_3_ cubic structure offers several benefits, such as being environmentally friendly and having a specific surface area, enhancing supercapacitor performance with a high specific surface area and an efficient mass transfer rate of electrolyte ions. The fabricated symmetric supercapacitor exhibited a higher *C*_Sp_ of approximately 212.5 F g^−1^ at 0.63 A g^−1^ with improved cyclic stability and excellent capacitance retention of about 99% after 5000 continuous cycles.^85^ Shereef *et al.* synthesized double perovskite La_2_NiMnO_6_ by the sol–gel technique. At a current density of 0.1 A g^−1^, the material exhibited a reduced specific capacity of 9.16 F g^−1^.^86^ Jose *et al.* reported the synthesis of La_2_FeMnO_6_, demonstrating high specific capacitance, good electrical conductivity, and long-term cycle stability. The mesoporous capacitance of La_2_FeMnO_6_ reached up to 10.9 mF g^−1^, with excellent capacitance retention of 96% after 5000 cycles.^87^

### 4.3 Solvothermal method

**Fig. 9:**
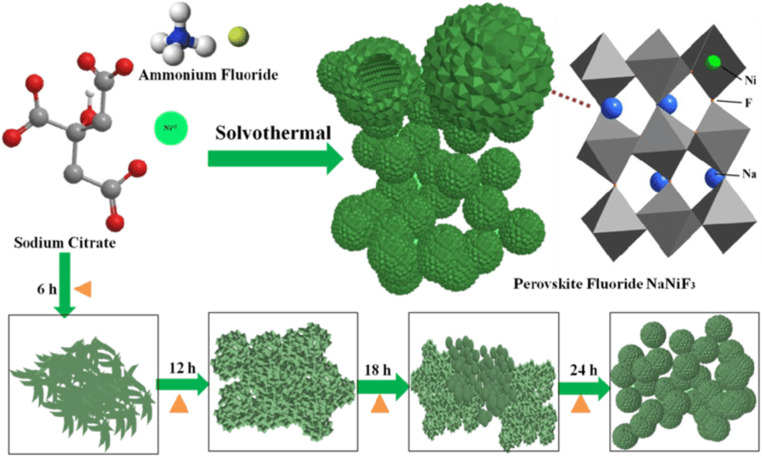
Synthesis process of hollow spherical NaNiF_3_ perovskite fluoride nanocrystals.^90^ This figure has been reproduced from ref. 90 with permission from Wiley, copyright 2025.

In the solvothermal technique, the solvent is nonaqueous, which is how it varies from the hydrothermal approach. On the other hand, hydrothermal synthesis produces the desired product by reacting precursors in a solution containing water, usually at high temperatures and pressure.^88^ Riaz *et al.* employed a solvothermal approach to produce KCdCl_3_/rGO and KCdCl_3_/C_60_. More reactive sites, efficient charge/ion movement, exceptional cycle stability, and high *C*_Sp_ are among the benefits provided by KCdCl_3_/C_60_ in electrochemical processes.^89^ Hussain *et al.* synthesized hollow spherical perovskite fluoride NaNiF_3_ using a one-step solvothermal process, as shown in Fig. 9. The asymmetric supercapacitor (SC) made with NaNiF_3_/AC electrodes demonstrated a high energy density of 51.78 W h kg^−1^ at 1.65 kW kg^−1^, a wide electrochemical window (1.65 V), and excellent cycling durability, maintaining 100% capacity retention from 1400 to 10 000 cycles. The production mechanism of hollow spherical perovskite fluoride has also been investigated.^90^ N. Bibi *et al.* hydrothermally produced SrZrO_3_ nanorods with high electrical conductivity, porosity, and cyclic stability. Therefore, the as-synthesized electrode demonstrates remarkable capacitance retention over 1000 cycles and a capacitance of 1225 F g^−1^ at a current density of 10 A g^−1^. Additionally, the fabricated electrode achieves a maximum power density of 4000 W kg^−1^ and an energy density of 65 W h kg^−1^.^91^

### 4.4 SILAR method

**Fig. 10:**
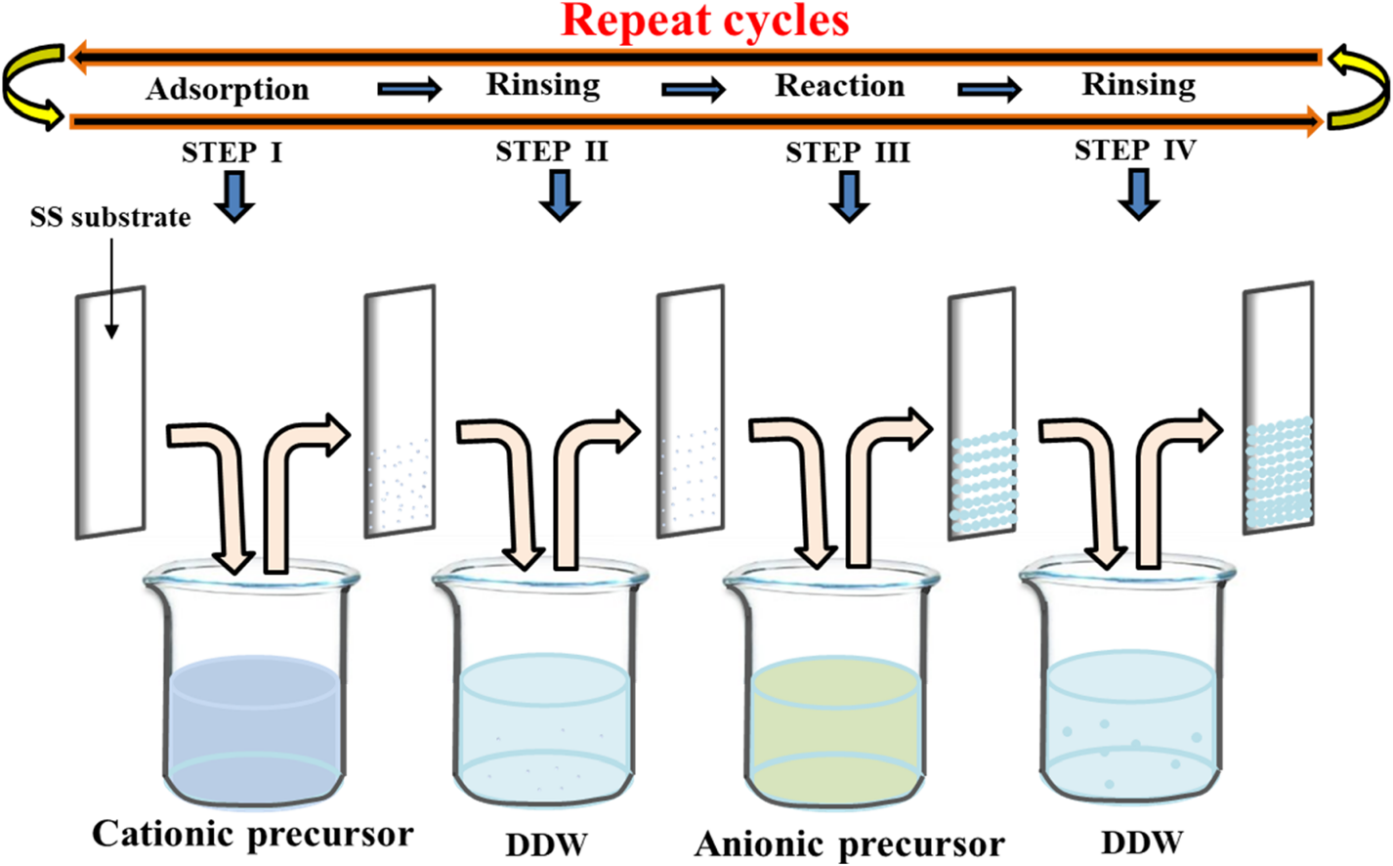
Schematic representation of SILAR method.^94^ This figure has been reproduced from ref. 94 with permission from Elseiver, copyright 2025.

SILAR is regarded as a relatively straightforward and economical technique for thin-film deposition, allowing for accurate control over thickness and composition.^92,93^ The method entails the repetition of four-step sequential adsorption, rinsing, reaction, and rinsing stages, leading to the layer-by-layer growth of thin films (Fig. 10). This layer-by-layer deposition mechanism enables precise film thickness and composition control, which can be fine-tuned by altering parameters such as precursor concentration, immersion time, and reaction conditions. In the 1980s, Nicolau *et al.* introduced the successive ionic layer adsorption and reaction (SILAR) method for fabricating thin films.^95^ Subsequently, a modified pseudo-SILAR (p-SILAR) method was developed for synthesizing doped and binary/ternary nanocomposites (NCs). Yasmeen *et al.* reported the synthesis of perovskite CsPb_2_Br_5_ nanocomposites using the SILAR method. It has been demonstrated that SILAR and p-SILAR are straightforward, cost-effective, rapid, and scalable synthesis techniques that involve exposing a substrate film or particles to ionic precursors, leading to (1) cation adsorption, (2) washing, (3) anion reaction, and (4) further rinsing.^96^ For instance, Ghule *et al.* synthesized nano pebbles-like BiVO_4_@C electrodes by the SILAR method. The following are the steps in the systematic method for the deposition of BiVO_4_@C: (1) the first beaker containing the cationic precursor (Bi(NO_3_)_3_ + BGBE) was submerged in the cleaned FSSM substrate for 10 s. During this time, Bi^3+^ ions were adsorbed onto the substrate due to the force of attraction between the ions in the solution and the substrate surface. (2) To eliminate the weakly bound Bi^3+^ ions, the FSSM substrate was submerged in the second beaker filled with double-distilled water for ten seconds. (3) In order to create stable BiVO_4_@C thin films, the previously adsorbed Bi^3+^ ions reacted with VO_4_^3−^ ions in the third beaker containing the anionic precursor (NH_4_VO_3_ + BGBE) for 10 seconds. (4) The substrate was rinsed with DDW for ten seconds in the fourth beaker to eliminate extra or unreacted species. During 5000 GCD cycles, the BiVO_4_@C electrodes exhibited enhanced cycling performance with 94.6% capacitive retention.^97^

### 4.5 Electrospinning method

**Fig. 11:**
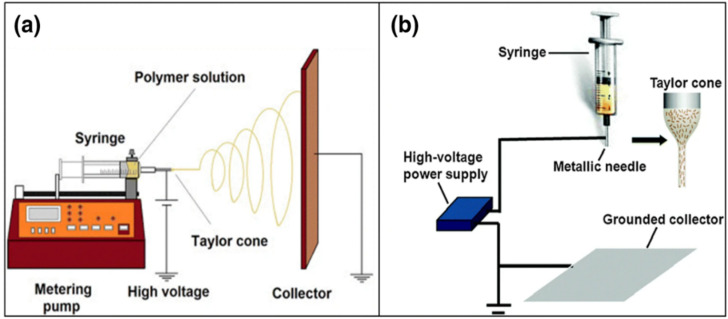
Schematic diagram of set up of electrospinning apparatus (a) horizontal set up and (b) vertical setup.^98^ This figure has been reproduced from ref. 98 with permission from Elseiver, copyright 2025.

Electrospinning is a complex yet cost-effective method to fabricate nanotubes and nanofibers with diameters ranging from several micrometres to tens of nanometers. Additionally, beyond the size and structure, it also depends on other factors such as the concentration of the parent compound, the viscosity of the parent compound solution, the type of polymer, and the electrospinning parameters, including voltage, working distance, and feed rate, which are other crucial factors (Fig. 11).^98^ In the case of supercapacitors, the perovskite-type metal oxide is a reliable electrode material with remarkable capacitive performance. Due to the high concentration of oxygen vacancies in these perovskites, they exhibit outstanding electrochemical properties. The supercapacitor based on La_*x*_Sr_1−*x*_NiO_3−*d*_ (0.3 ≤ *x* ≤ 1) NFs demonstrated exceptional electrochemical characteristics, achieving 719 F g^−1^ at 2 A g^−1^.^99^ Similarly, La_*x*_Sr_1−*x*_Co_0.1_Mn_0.9_O_3−*δ*_ (0.3 ≤ *x* ≤ 1) NFs demonstrated a capacitance of 485 F g^−1^ at 1 A g^−1^ in 1 M KOH. Similarly, the supercapacitive performance of SrMnO_3_ NFs has been investigated concerning the effects of doping Ba, Ca, and Ni on the Sr site and Co, Fe, and Ni on the Mn site, respectively. The study revealed that adding 20 mol% Ba to the SrMnO_3_ matrix enhanced capacitance from 321.7 F g^−1^ to 446.8 F g^−1^ at 0.5 A g^−1^.^100^

## 5. Strategies of perovskite oxides on the performance of supercapacitors

### 5.1 A-Site doping

Generally, the supercapacitor performance of perovskite oxides is directly linked to the B-site transition metal element. The A-site component, which consists of alkaline earth and lanthanide elements, is typically inert in the redox reaction. However, the cation at the A-site may influence the electronic coordination and structure. A significant improvement occurs when a low-valence cation partially replaces the A-site, causing some B-site transition-metal ions to shift to unstable oxidation states (B^*m*+^/B^(*m*+1)^)^+^ redox pair, such as Sr^2+^ and Ca^2+^. This alteration also generates additional oxygen vacancies. Consequently, the electrochemical performance is enhanced, and electronic conductivity increases. It is environmentally advantageous for alkaline earth metals to replace rare earth metals due to their similar atomic radii. In this context, Roy *et al.* utilized a sol–gel technique to synthesize Ca-doped perovskite lanthanum manganates (La_0.5_Ca_0.5_MnO_3_). The *C*_Sp_ of La_0.5_Ca_0.5_MnO_3_ was 2.4 times higher than that of pure LaMnO_3_.^101,102^ Similarly, Wang *et al.* doped LaMnO_3_ with Sr and observed that after 1000 cycles, the cycle life enhanced from 40% to 80%, and the capacitance slightly increased from 187 to 198 F g^−1^ at 0.5 A g^−1^. Meanwhile, Tian *et al.* used the sol–gel method to synthesize La_1−*x*_Sr_*x*_MnO_3_ (*x* = 0, 0.15, 0.3, 0.5) to understand better how the substitution degree influences electrochemical performance.^103^ It was discovered that the value of *x* influences the charge transfer resistance, *C*_Sp_, and degree of nanoparticle aggregation.^103^ A group of PO with the composition La_1−*x*_Sr_*x*_BO_3−d_ (*x* = 0–1; B = Fe, Mn, Co) was recently synthesized by Wang *et al.* to investigate anion-based pseudocapacitors systematically. They found that the surface-normalized capacity, whose slope is determined by the B-site element, increases linearly with a higher oxygen vacancy content following the systematic addition of Sr^2+^.^102^ Compared to the Fe and Co oxides, La_0.2_Sr_0.8_MnO_2.7_ exhibited the highest *C*_Sp_ of approximately 492 F g^−1^. The energy required for the aliovalent substitution of oxygen vacancies depends on the alkaline-earth metals. Luo *et al.* investigated the influence of Ba and Ca doping on SrMnO_3_ nanofibers. By doping the SrMnO_3_ matrix with 20 mol% Ba, the *C*_Sp_ significantly increased from 321.7 F g^−1^ to 446.8 F g^−1^. Furthermore, the Sr_0.8_Ba_0.2_MnO_3_ based ASC demonstrated excellent capacitance retention of 87% after 5000 cycles and an energy density of 37.3 W h kg^−1^ at a power density of 400 W kg^−1^.^104^

### 5.2 B-Site doping

**Fig. 12:**
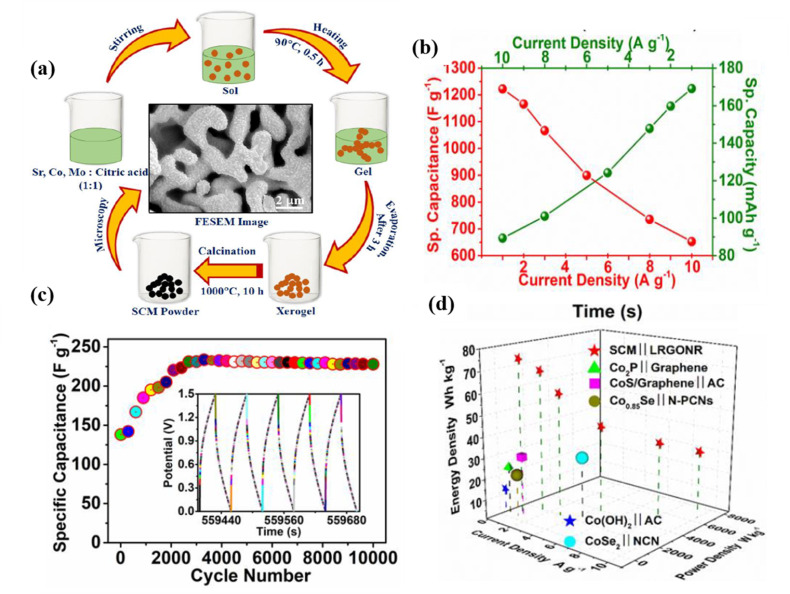
(a) Schematic representation of the synthesis process of SCM. (b) GCD curves of the SCM electrode at varying current densities. (c) Cycling stability of the ASC at 10 A g^−1^. The inset displays the final five GCD cycles. (d) Ragone plot of a hybrid SCM cell compared with literature findings.^98^ This figure has been reproduced from ref. 98 with permission from Elseiver, copyright 2025.

Cobalt-based PO exhibit greater efficiency than Mn-based ones due to their enhanced oxygen-ion mobility and higher concentration of oxygen vacancies. Certain cobalt-based PO such as cubic phase, at room temperature SrCoO_3−*δ*_, are unstable. Sharma *et al.* partially substituted Mo for Co in SrCoO_3−*δ*_ to widely deploy cobalt-based PO in SCs (Fig. 12a).^34^ It was found that SrCo_0.9_Mo_0.1_O_3_ (SCM) had 2.1 times more oxygen vacancies than SrCoO_3−*δ*_. Furthermore, compared to SrCoO_3−*δ*_, SCM exhibits a *C*_Sp_ of approximately 1223.34 F g^−1^ at 1 A g^−1^ (Fig. 12b). Notably, after 10 000 cycles, the ASC, utilizing lacey-reduced graphene oxide nanoribbon (LRGONR) as the negative electrode, demonstrated long-term stability (Fig. 12c). At a power density of 734.5 W kg^−1^, it also achieved a specific energy density of 74.8 W h kg^−1^ (Fig. 12d).^105^ Similarly, Shao *et al.* reported Nb-doped SrCoO_3−*δ*_ with a gravimetric capacitance of approximately 773.6 F g^−1^ and excellent cycling stability, exhibiting about 95.7% capacitance retention after 3000 cycles. Additionally, an ASC was assembled with AC and SrCo_0.9_Nb_0.1_O_3−*δ*_ (SCN), serving as the cathode and anode, respectively. When the power density was 433.9 W kg^−1^, the device's energy density reached 37.6 W h kg^−1^, and when the power density increased to 9864.2 W kg^−1^, it continued to maintain an energy density of 32.9 W h kg^−1^. Furthermore, the potential window of PO may be influenced by B-site doping.^64^ G. Singh *et al.*^105^ explored the impact of B-site element doping on the stability window of SrRuO_3_. Notably, it was found that doping SrRuO_3_ with 20 mol% Mg enhanced its *C*_Sp_ without altering its stability window. Conversely, substituting Fe or Co could lead to a reduced stability window.

### 5.3 Modulating stoichiometry

**Fig. 13:**
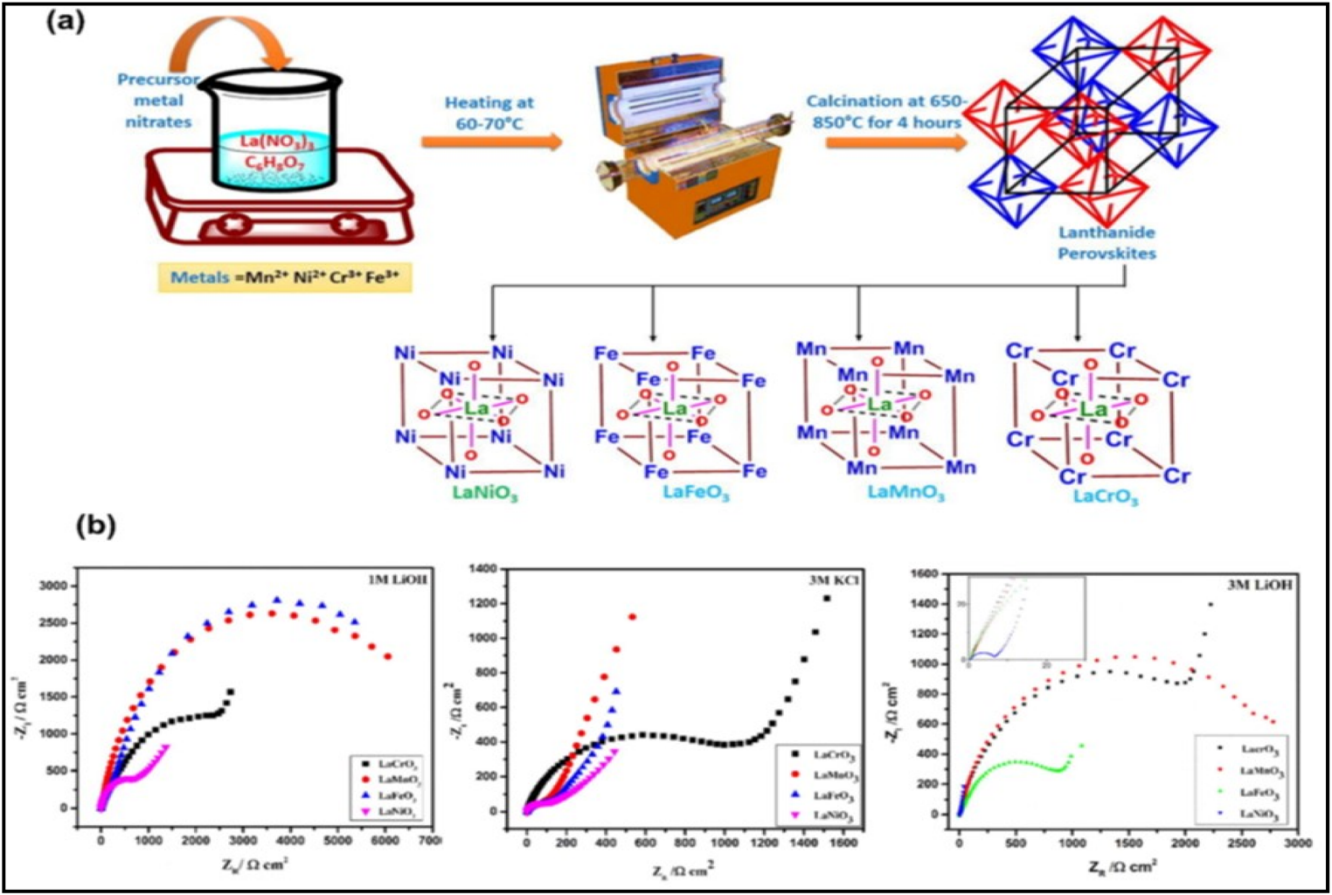
(a) Synthesis procedure for four types of different perovskite oxide powders (LaMnO_3_, LaFeO_3_, LaCrO_3_, and LaNiO_3_) for application as SC anode materials using the sol–gel method. (b) EIS of the four perovskite materials in various electrolyte solutions.^107^ This figure has been reproduced from ref. 107 with permission from Elseiver, copyright 2025.

Various post-processing techniques, including heat treatment at elevated temperatures in an environment with low oxygen partial pressure and inert conditions, are used in a reducing atmosphere like H_2_ to produce oxygen-deficient POs with the general formula ABO_3−*δ*_. According to research by Mefford *et al.*, sub-stoichiometric LaMnO_2.91_ performed better than anion excess form LaMnO_3.09_.^106^ Oxygen vacancy concentration and overall performance are also significantly impacted by cation deficiencies. The influence of cation stoichiometry in LaMnO_3_ was explored by Qian *et al.* by manipulating the A and B-site cation ratio. The LaMn_1±*x*_O_3−*δ*_ outperformed the stoichiometric LaMnO_3_. Although supercapacitor performance was not specifically examined, perovskites with A-site cation deficiency have been demonstrated to produce oxygen-deficient structures.^107^Fig. 13 shows the synthesis process of different perovskite oxide powders.

## 6. Design of perovskite electrode materials

Design principles for perovskite electrode materials can be clarified based on the charge storage mechanism of the perovskite oxide electrode. These principles can be classified into the following sections.

### 6.1 Creating oxygen vacancy

According to the perovskite oxide structure theory, substituting the A site with a low valence can enhance the valence of the B site element or increase the oxygen vacancy concentration.^108^ The oxygen vacancy concentration is only marginally influenced by replacing the A site with a high valence, which may reduce the valence of the B or A sites. As the concentration of oxygen vacancies increases, more hydroxide anions intercalate through the electrode surface and diffuse from the electrolyte to the electrode more efficiently.^109^ However, elevating the B site element's valence restricts the theoretical *C*_Sp_. Consequently, enhancing the electrochemical performance of perovskite by replacing the A site with low valence is an inefficient technique. Even though achieving theoretical specific capacitance is highly challenging, the approach can enhance electrochemical performance due to its partially diminished internal resistance and capability to facilitate diffusion and intercalation quantity.^110–112^ The internal resistance of perovskite, which affects the electrode's power density, is influenced by the B–O–B bond length and angle, which are determined by the atomic size and the type of doped element. While certain perovskite structures have been identified, accurately measuring the crystal structure's B–O–B bond angle and length remains a significant challenge.^113^ These bond angles and lengths require first-principles simulations for a more precise explanation. The relationship between bond angle, length, and doped element can be analyzed through perovskite material design in conjunction with first-principles simulations. Additionally, it can calculate the theoretical internal resistance of perovskite, which may help reduce the development cycle of doped perovskite design.^114^

In conclusion, *C*_Sp_ and power density can be optimized by developing element-doped perovskite oxides with high oxygen vacancies and minimal internal resistance. Several examples of suitable composition selection are provided, including Ti, Nb, Mo, and V, whose oxides have been used as electrodes to fabricate electrochemical capacitors. With high valence states, these elements doped in the B site can enhance the oxygen vacancy in perovskites.^115,116^

### 6.2 Designing the microstructure and high specific surface area

A large specific surface area can enhance the *C*_Sp_ and ion exchange rate between electrodes and electrolytes. The advantages of different microstructures for supercapacitors vary.^117,118^ A core–shell structure can improve the electrode's cycle stability by limiting the core material's phase transition. The electrode's resistance can be reduced by shortening the electron transfer path from the active material's surface to the current collector using a nanoneedle or nanosheet structure. Perovskites with various nanostructures are expected to exhibit higher *C*_Sp_, longer cycle stability, greater power density, and increased energy density.^119,120^ However, there is still much to discover due to different microstructures were created through hydrothermal reactions, guiding agents like urea, hexamine, and ammonium hydroxide, which stop hydroxide coprecipitation from happening when lanthanum-based perovskites are being prepared. Although the sol–gel method can produce flower-like and nanowire perovskites, these structures are unsuitable for *in situ* growth on the surface of the current collector.^121,122^ Therefore, to achieve successive coprecipitation or obtain perovskite *in situ*, further research should focus on developing a novel guiding agent for the hydrothermal reaction of perovskite. Binder-free *in situ* growth can improve the electrochemical performance of perovskites by preventing issues related to poor conductivity.^123,124^

First, at the perovskite calcination temperature, conventional current collectors such as carbon paper and nickel foam undergo significant oxidation in the air. Therefore, protection with argon or nitrogen is necessary. Second, the formation of perovskite is impeded by certain transition metal hydroxides, like Mn(OH)_2_, Ni(OH)_2_, and Co(OH)_2_, which can only be converted into diatomic metal oxides and cannot undergo further oxidation when protected by nitrogen or argon.^125^ Consequently, it is essential to prepare the trihydroxide before calcination. As a result, Fe and Ti, which can exist as Fe(OH)_3_ and Ti(OH)_3_, are promising candidates for *in situ* perovskite production. In conclusion, hydrothermal reactions are necessary to produce perovskites with a wider range of microstructures, but careful selection of guiding agents and B-site components is essential to obtain the desired perovskites.^126^

### 6.3 Developing perovskite composites

Three main components comprise the design concept for perovskite composites: initially, perovskites can be combined with oxides whose potential window partially overlaps the perovskite's potential window. Furthermore, this kind of perovskite composite can enhance its integral potential window.^127,128^ At the same time, the energy density of composites is enhanced when the specific capacitance is either minimally affected or even increased by the extended potential window. Additionally, perovskites can be combined with oxides with a different redox potential than the perovskites themselves. This enables simultaneous charge storage at competing active sites during the charging process, enhancing the specific capacitance. The combination of La_0.85_Sr_0.15_MnO_3_ with NiCo_2_O_4_ was recently explored, addressing the challenge of binder-free electrodes for perovskites with a core–shell architecture. This material demonstrated remarkable electrochemical performance, including a high specific capacity of 260.75 mA h g^−1^ at 0.5 A g^−1^ in 6 M KOH. Additionally, it demonstrated exceptional cycle stability, retaining 200% after 10 000 cycles at a current density of 20 A g^−1^. When incorporated into hybrid supercapacitors, it achieved an energy density of 63.5 W h kg^−1^ at a power density of 900 W kg^−1^.^129^ The high electrochemical performance can be attributed to the synergistic interaction of Mn, Co, and Ni, coupled with the excellent contact between the active materials and the current collector. Lastly, perovskites can be combined with a conductor to form a composite. Even though perovskites have good electrical conductivity, most cannot match conductor conductivity. Combining perovskites with conductors can further enhance electrochemical performance due to resistance affecting power density and SpC.^130^

## 7. Applications of perovskite oxides towards supercapacitors

Previous studies suggest that the defective structure of perovskite oxide and its enhanced oxygen ion mobility can facilitate the conversion of B^*x*+^ to a high valence state, thereby improving electrochemical properties. However, limitations of PO, such as their restricted surface area and high transport resistance of aggregated nanoparticles, impede further enhancement of electrochemical performance.^131,132^ Combining ABO_3_ with other materials, such as metal oxides, carbon materials, and noble metals, to form composites with enriched chemistry effectively solves these challenges.

### 7.1 Perovskite oxide based electrode materials

#### 7.1.1 Strontium-based perovskite oxides

Strontium-based perovskite oxides have attracted considerable research attention as supercapacitor electrode materials due to their natural abundance, excellent ionic and electronic conductivity, and resistance to redox cycling. Table 1 compares the specific capacitance of various strontium-based PO. SrFeO_3_, SrCoO_3_, and SrCoFeO_3_, representing three distinct perovskites, were synthesized using the sol–gel method, achieving a high density of oxygen vacancies and exceptional capacitance. Co-doping with a 1 : 1 ratio (Co/Fe) at the B site of SrFeO_3_ decreased the *C*_Sp_ when applied to supercapacitors. The synthesized electrode demonstrated a *C*_Sp_ of approximately 60.912 F g^−1^ at a scan rate of 10 mV s^−1^. Additionally, nitrogen adsorption and desorption measurements were conducted to gain a detailed understanding of the porosity characteristics of the synthesized samples. SEM images revealed a rough and highly porous structure that enhances the electrode's active surface area, consistent with theoretical values.^136^ George *et al.* synthesized SrMnO_3_ perovskite oxide nanofibers using a sol–gel electrospinning method followed by calcination at various temperatures.^100^ The resulting electrode exhibited enhanced electrochemical properties, including a higher specific capacitance, excellent rate capability (446.8 F g^−1^ at 0.5 A g^−1^), improved cyclic stability (87% at 5000 cycles), and extended cycle life. The device fabricated with these nanofibers demonstrated a specific energy of 37.3 W h kg^−1^ at a specific power of 400 W kg^−1^, proving its effectiveness as an electrode material for high-rate charge–discharge performance in supercapacitors. Lei *et al.* synthesized SrCoTiO_3_ using a solid-state reaction method. However, it exhibited the major drawback of poor electrochemical activity as an electrode material with *C*_Sp_ of 114.4 F g^−1^.^133^ Using the solid-state reaction method, Lei *et al.* synthesized SrBNbO_3_ (where B = Mn, Co). The subsequent electrochemical performance of an asymmetric supercapacitor demonstrated a higher *C*_Sp_ of about 894 F g^−1^ at 1 A g^−1^ with a capacitance retention of 88.88% after 10 000 cycles.^133^

**Table 1:** Comparison of specific capacitance of various strontium based perovskite oxides

Electrode material	Synthesis method	Specific capacitance (F g^−1^/mF cm^−1^)	Ref.
Sr_2_CoMoO_6−*δ*_	Sol–gel method	747	105
SrCo_0.875_Nb_0.125_O_3_	Solid state reaction	894	133
SrCo_0.9_Nb_0.1_O_3−*δ*_	Solid state reaction	774	133
Ni–SrTiO_3_	Ball milling method	142	134
Sr_2_CoSbO_6_	Solid-state method	228	135

#### 7.1.2 Lanthanum-based perovskite oxides

**Fig. 14:**
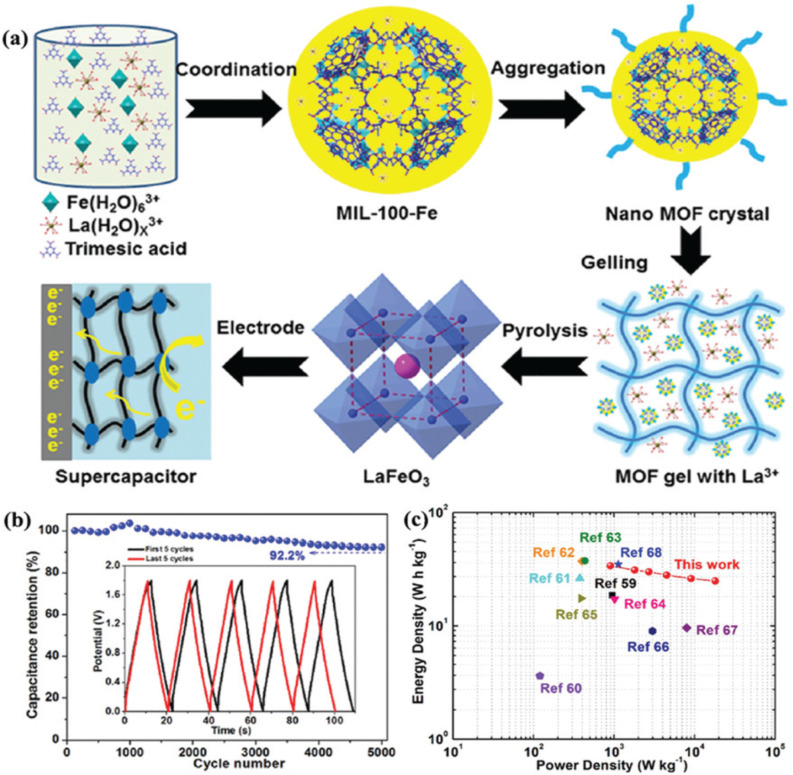
(a) Schematic representations illustrating the synthesis of perovskite LaFeO_3_. (b) Graph showing cycling stability *versus* cycle number, inset: the first and last five GCD cycles. (c) Ragone plots (energy density *vs.* power density) of this study and other devices at 2 A g^−1^.^138^ This figure has been reproduced from ref. 138 with permission from RSC, copyright 2025.

Lanthanum-based perovskite oxides have gained significant attention in supercapacitors due to their high thermal stability, ease of synthesis, oxygen storage capacity, low cost, and excellent electrical conductivity. LaMnO_3_ is the first lanthanum-based perovskite oxide to be employed in SCs. The lattice, which is deficient in cations and the presence of manganese in two oxidation states (Mn^3+^/Mn^4+^), leads to a stable and consistent oxygen excess in LaMnO_3_.^137^Table 2 compares the specific capacitance of various lanthanum-based perovskite oxides. Augustyn *et al.* reported a specific capacitance of 609.8 F g^−1^ for LaMnO_3_.^171^ LaNiO_3_ exhibits a charge storage mechanism similar to that of LaMnO_3_ but demonstrates lower electrical resistivity (approximately 10^−4^ Ohm). Shao *et al.* employed a template-free solvothermal method to synthesize a hollow spherical structure of LaNiO_3_, leading to a high specific capacitance (422 F g^−1^ at 1 A g^−1^) and excellent cycle stability (83.3% capacitance retention after 5000 cycles).^172^ Compared with LaNiO_3_, LaFeO_3_ is more stable because Fe^3+^ has a stable electronic configuration 3d^5^. Zhang *et al.* used mesoporous LaFeO_3_ nanoparticles as an electrode material for SCs (Fig. 14a). The fabricated symmetric SC cell (SSC) exhibits a high energy density of 34 W h kg^−1^ and a power density of 900 W kg^−1^, retaining 92.2% capacitance after 5000 cycles (Fig. 14b and c).^138^ Harikrishnan *et al.* used coprecipitation to create LaCoO_3_ nanoparticles. LaCoO_3_ has good electrochemical redox properties due to its multiple oxidation states (+2, +3 and +4), with a *C*_Sp_ of 299.64 F g^−1^ at 10 A g^−1^.^164^ Hussain *et al.* synthesized a hierarchical mesoporous nanostructure of LaCrO_3_*via* the sol–gel method. The electrode exhibits a high *C*_Sp_ of 1268 F g^−1^.^156^

**Table 2:** Comparison of specific capacitance for various lanthanum-based perovskite oxides

Electrode material	Synthesis method	Specific capacitance (F g^−1^/mA h g^−1^)	Electrolyte	Ref.
LaFeO_3_	Sol–gel	241	1 M Na_2_SO_4_	138
La_2_NiFeO_6_	Solvothermal	768	2 M KOH	139
La_0.2_Sr_0.8_MnO_2.7_	Co-precipitation	492	1 M KOH	140
LaMnO_3_	Co-precipitation	520	0.5 M Na_2_SO_4_	141
La_0.8_Na_0.2_Fe_0.8_Mn_0.2_O_3_	Modified Pechini	56	1 M H_2_SO_4_	141
La_0.8_Sr_0.1_5MnO_3_@NiCo_2_O_4_	Hydrothermal	1341	6 M KOH	142
LaMnO_3_/Mn_3_O_4_	One pot	135	1 M Na_2_SO_4_	143
La_1−*x*_Sr_*x*_MnO_3_	Sol–gel	102	1 M KOH	144
La_*x*_Sr_1−*x*_NiO_3−*δ*_	Electrospinning	719	1 M Na_2_SO_4_	145
LaNi_1−*x*_Fe_*x*_O_3−*δ*_	Modified Pechini	894	1 M KOH	146
LaNiO_3_	Electrospinning	116	6 M KOH	147
La_2_ZnMnO_6_	Hydrothermal	718	2 M KOH	148
La_2_CuMnO_6_	Hydrothermal	205	2 M KOH	149
La_2_FeCoO_6_	Sol–gel	831	2 M KOH	150
La_2_CoMnO_6_	Impregnation	376	1 M Na_2_SO_4_	151
La_0.5_Ca_0.5_MnO_3_	Sol–gel	170	1 M KOH	152
LaNiO_3_	Solvothermal	422	6 M KOH	153
La_0.7_Sr_0.3_FeO_3_	Electrospinning	523	1 M Na_2_SO_4_	154
La_2_CoNiO_6_	Solvothermal	635	2 M KOH	155
LaCrO_3_	Sol–gel	1268	1 M LiCl	156
LaFe_0.5_Cr_0.5_O_3_	Sol–gel	16	6 M KOH	157
La_0.85_Sr_0.15_Mn_0.9_Ni_0.1_O_3_	Electrospinning	113	1 M KOH	158
La_0.7_Sr_0.3_Co_0.1_Mn_0.9_O_3−*δ*_	Electrospinning	485	1 M KOH	159
Ag/La_0.7_Sr_0.3_CoO_3−*δ*_	Ball milling	517	1 M KOH	160
LaNiO_3_	Sol–gel	139	6 M KOH	161
La_0.7_Sr_0.3_MnO_3_	Ball milling	393	1 M Na_2_SO_4_	162
La_1−*X*_Ag_*X*_MnO_±*δ*_	Co-precipitation	152	6 M KOH	163
LaMnO_3_@NiCo_2_O_4_	Hydrothermal	811	6 M KOH	159
LaCoO_3_	Co-precipitation	299	3 M KOH	164
La_0.8_Nd_0.2_Fe_0.8_Mn_0.2_O_3_	Hydrothermal	158	3 M KOH	165
LaCoO_3_	Plasma etching	706	6 M KOH	165
La_0.7_Sr_0.3_CoO_3−*δ*_@MnO_2_	Electrospinning	570	6 M KOH	165
LaSr_0.85_Mn_0.15_O_3_	Sol–gel	198	1 M KOH	166
La_2_CoNiO_6_	Electrospinning	335	6 M KOH	167
La_0.6_Sr_0.4_NiO_3−*δ*_	Sol–gel	115	6 M KOH	168
La_2_NiO_4+*δ*_	Citrate method	657	3 M KOH	169
La_1−*X*_K_*X*_FeO_3−*δ*_S	Ceramic synthesis	662	2 M KOH	170
LaFeO_3_	Electrospinning	183	6 M KOH	169
LaCoO_3_	Electrospinning	95	6 M KOH	169

#### 7.1.3 Cerium-based perovskite oxides

Cerium-based POs are promising because of their high dielectric constant, low cost, high bandgap, and variable valence states (Ce^3+^ and Ce^4+^).^170^ For instance, Nsar *et al.* employed electrospinning followed by calcination processes to synthesize CeMnO_3_ nanofibers (NFs).^173^ It is widely accepted that A-site cations of PO do not contribute to the electronic structure near the Fermi level. However, due to cerium's high redox capability between Ce^3+^ and Ce^4+^, the faradaic redox reaction (Ce^4+^/Ce^3+^ and Mn^3+^/Mn^4+^) occurred on the CeMnO_3_ electrode surface. CeMnO_3_ nanofibers exhibits *C*_Sp_ of 159.59 F g^−1^ at 1 A g^−1^ current density. Cerium-based POs (CeCoO_3_, CeNiO_3_, and CeCuO_3_) have recently shown *C*_Sp_ of 128, 189, and 117 F g^−1^, respectively.^174^ Ahangari *et al.* compared the electrochemical properties of CeMO_3_(M = CO, Ni, Cu), among which CeNiO_3_ nanoplates shows high *C*_Sp_ of 189 F g^−1^ with good cyclic stability.^175^ Harikrishnan *et al.* synthesized by coprecipitation method, and a symmetric supercapacitor was fabricated with the prepared material showing energy and power density 27 W h kg^−1^ and 826 W kg^−1^ with better cyclic stability of 92% at 5000 cycles.^176^

#### 7.1.4 Calcium-based perovskite oxides

CaTiO_3_, a perovskite oxide material, is attracting interest for future applications in supercapacitors. However, pure CaTiO_3_ is not commonly used directly as an electrode material in supercapacitors due to its comparatively low electrical conductivity. Researchers focused on doping or mixing its electrochemical performance with other substances to enhance its electrochemical performance.^177,178^ Lang *et al.* investigated CaTiO_3_ combined with Activated Carbon (AC) to enhance the specific surface area and electrochemical performance of supercapacitors. The CaTiO_3_-AC composite showed a *C*_Sp_ of 270 F g^−1^, significantly higher than pure CaTiO_3_. The activated carbon contributed to a higher surface area and better ion adsorption, improving the energy storage capacity. The composite also exhibited good long-term cycling stability.^179,180^

### 7.2 Perovskite oxides based composite electrode materials

#### 7.2.1 Perovskite oxides with noble metals

The movement of electrons produced by the oxidation or reduction of PCs to the current collectors can be facilitated by noble metals, such as platinum (Pt) and gold (Au), which have good electric conductivity.^181^ However, because noble metals are expensive and scarce, combining them with other affordable and sustainable materials is one of the most appealing ways to reduce their use.^182,183^ One advantage of PO is that they are naturally occurring and reasonably priced. Unfortunately, PO still lack sufficient electrical conductivity. Consequently, the synergistic effects of combining PO and noble metals are anticipated to enhance their SC performance. Ag is the noble metal with the highest conductivity.^184^ Moreover, it offers the benefit of an acceptable activity and a reasonable cost. Cao *et al.* synthesized an Ag nanoparticle decorated La_0.85_Sr_0.15_MnO_3_ and employed it as an electrode for SC. It can create electron transfer channels because silver has a far higher electrical conductivity than carbon. The redox reaction between Ag and Ag_2_O in an alkaline electrolyte solution may also slightly influence pseudocapacitance. The Ag@LSM15 composite thus produced a long cycle life retaining 100% capacitance retention after 1000 cycles and a high *C*_Sp_ of 186 F g^−1^ at 1 A g^−1^.^185,186^ Another study used a porous perovskite La_0.7_Sr_0.3_CoO_3−*δ*_ (LSC) substrate (Ag/LSC) to grow Ag nanoparticles directly. The performance was examined with varying mass loadings of Ag of about 10.61, 30.60, and 51.31 mg. When the Ag content was 30 mg (30 Ag/LSC) or less, the surface became rough, and the porous structure of LSC was maintained. This advantageous structure may make more active surface sites and quicker mass transport possible. In addition, the Ag/LSC electrode with 30 mg Ag loading showed the best performance of 14.8 F cm^−2^ due to lower *R*_s_ of 1.28 Ω cm^2^ and *R*_ct_ of 0.61 Ω cm^2^.^179^

#### 7.2.2 Perovskite oxides with metal oxides

Due to their numerous oxidation states, metal oxides have drawn increasing attention because they can store energy up to an order of magnitude more generously than carbon-based EDLCs. However, during charge/discharge processes, most metal oxides have poor durability, low conductivity, and poor rate capability. On the other hand, the stable structure of PO allows for improved surface oxygen exchange kinetics and significantly higher oxygen ion/electron conductivity.^185^ Since being inexpensive and having the potential to achieve high *C*_Sp_/capacity values, exhibiting exceptional stability, perovskite oxide and metal oxide composites are attractive alternatives. Among the metal oxides, MnO_2_ is unique because of its natural abundance, low cost, and high theoretical *C*_Sp_ of roughly 1370 F g^−1^. As an electrode for SCs, Jingbo *et al.* used a hydrothermal method to create a ((La_0.75_Sr_0.25_)_0.95_MnO_3−*δ*_(LSM)/MnO_2_) composite. The resulting electrode exhibits a higher *C*_Sp_ of about 437.2 F g^−1^ at 2 mV s^−1^.^186^ Although CeO_2_ has distinct chemical characteristics, it has a lower theoretical capacitance than MnO_2_. It is, therefore, readily oxidized and reduced throughout the oxidation-reduction process. LaMnO_3_ mixed CeO_2_ (CeO_2_/LaMnO_3_) nanocomposites with a greater *C*_Sp_ of about 262 F g^−1^ at 1 A g^−1^ in 1 M Na_2_SO_4_ solution were reported by Nagamuthu *et al.* The CeO_2_/LaMnO_3_ nanocomposite worked better with ASC-negative electrodes. In particular, an ASC device was constructed with AC as the positive electrode and CeO_2_/LaMnO_3_ nanocomposites as the negative electrode. This resulted in an energy density of 17.2 W h kg^−1^ at a power density of 1015 W kg^−1^.^187,188^ The relationship between the potential window (*V*) and energy density (*E*) is well known. Therefore, expanding the potential window is an additional method of raising the energy density. Stoller *et al.* synthesized La_0.8_Sr_0.15_MnO_3_@NiCo_2_O_4_ (LSM15@NC) core–shell nanoflower structure grown directly on Ni foam. LSM15@NC displayed a broad window and the coexistence of PC and EDLC behaviour. Moreover, the ASC produced an energy density of 63.5 W h kg^−1^ at a power density of 900 W kg^−1^ when the AC was used as the negative electrode and the LSM15@NC composite as the positive electrode.^189^

#### 7.2.3 Perovskite oxides with carbonaceous materials

Carbonaceous materials with a large specific surface area, good electronic conductivity, and high chemical stability, such as graphene, reduced graphene oxide, graphene, and AC, have been used extensively in SC. The low intrinsic conductivity of perovskite oxide limits their usage in SC. An efficient method to address this shortcoming is, incorporating carbonaceous materials to create PO/carbon composites. To improve SC performance, graphene is typically added to perovskite oxides because of its superior electrical conductivity of 6000 S cm^−1^ and extra-large theoretical specific surface area of 2630 m^2^ g^−1^.^186^ Agglomeration of perovskite oxides can be effectively inhibited by graphene. It can offer a fast channel for the transport of electrons in the meantime. The loading of perovskite oxide nanoparticles increases the distance between graphene sheets; they can also preserve the structural integrity of monolayer graphene. Immobilized BiFeO_3_ (BFO) nanowires on nanometer-thin RGO show superior charge transfer resistance and *C*_Sp_ of about 368.28 F g^−1^ compared to BFO and RGO. The electrolyte also significantly affects BFO-RGO performance, which is noteworthy.^190^ To examine the capacitive behaviour of graphene–perovskite oxide compound materials in aqueous electrolytes with varying basicity or acidity, Jingbo *et al.*^186^ tested reduced graphene performance sheets decorated SrRuO_3_ (SRGO) in three different electrolytes: 1.0 M KOH, 1.0 M NaNO_3_, and 1.0 M H_3_PO_4_. The SRGO showed the highest capacitance of 160 F g^−1^ in 1.0 M KOH. Adding carbon improves the electrochemical performance compared to pure PO. Pseudocapacitance is caused by the redox reaction of oxygenated groups on the surface of carbon nanostructures. Accordingly, adding heteroatoms or surface functional groups to the surface of carbon-based materials is a useful method of raising the capacitance of the composite electrode. Cheng *et al.* introduced a heteroatom to reduce graphene oxide (rGO) by substituting the hydroxyl groups with the nitrogen atoms. A three-dimensional network (LMO/N-rGO) can then be created by directly integrating the as-prepared nitrogen-doped graphene (N-rGO) sheets with LaMnO_3_ (LMO) *via* electrostatic interactions. The resulting nanocomposites showed the best stability of 79% capacitance retention after 2000 cycles at 10 A g^−1^ and a *C*_Sp_ of 687 F g^−1^ at 5 mV s^−1^ compared to pristine graphene and LMO.^191^ In a different study, Shafi *et al.* used *in situ* chemical polymerization to create a composite material comprising LaMnO_3_, RGO, and polyaniline (PANI). Due to the excellent structural stability and electrical conductivity offered by the RGO support and PANI coating, the synthesized ternary composite demonstrated a *C*_Sp_ of 802 F g^−1^ at 1 A g^−1^.^192^

#### 7.2.4 Perovskite oxides with conducting polymers

**Fig. 15:**
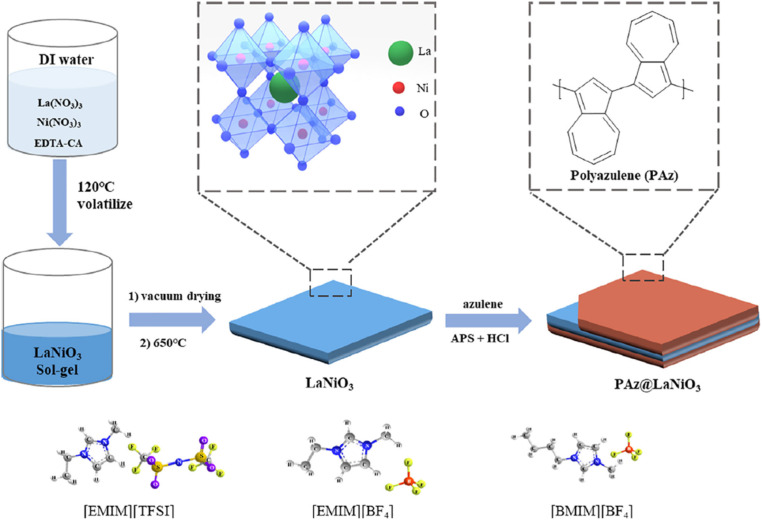
Schematic representation of the preparation of PAz@LaNiO_3_ nanosheets.^195^

Riaz *et al.* reported the synthesis of KCuCl_3_-polyaniline (PANI) composites. KCuCl_3_-polyaniline (PANI) composites have excellent electrochemical properties, with *C*_Sp_ values as high as 1757 F g^−1^. This high-performance is due to the positive relationship of KCuCl_3_ conductivity and structural integrity with PANI's pseudocapacitive activity. These composites are good options for advanced supercapacitor applications due to their enhanced mechanical durability, cyclic stability, and energy storage efficiency.^193^ According to the study, this improved performance was achieved by the conducting polymer's effective result with CaTiO_3_'s strong dielectric properties, which improved cycle stability and charge storage. At a current density of 6.86 A g^−1^, the composite showed a *C*_Sp_ of 984.21 F g^−1^. The power density was 3.2 kW kg^−1^, and the energy density was 58.14 W h kg^−1^.^194^ LaNiO_3_ nanosheets and polyazulene are promising materials for supercapacitors due to their electrochemical properties. LaNiO_3_, a perovskite oxide, exhibits high capacitance and fast charge/discharge cycles when synthesized into nanosheet form, as shown in Fig. 15. The nanosheet morphology increases surface area, improving electrochemical performance. Polyazulene, an organic conductive polymer, offers good conductivity and redox activity, enhancing charge storage. It undergoes reversible electrochemical reactions, making it suitable for supercapacitors. Combining LaNiO_3_ nanoparticle nanosheets with polyazulene in composite electrodes exhibits improved capacitance and cycling stability. It exhibited a *C*_Sp_ of 464 F g^−1^ at a current density of 2 A g^−1^. Thus, the material serves as a promising candidate for advanced energy storage devices.^195^

This figure has been reproduced from ref. 195 with permission from Wiley, copyright 2025.

## 8. Conclusion and future outlooks

Perovskite oxides (POs) have recently gained widespread attention as electrode materials for supercapacitors (SCs) due to their unique structure, compositional flexibility, and inherent oxygen vacancy. Notably, PO, as an active component in intercalation-type capacitors, possess high concentrations of oxygen vacancies and do not require significant surface area for energy storage. This article primarily compiles the recent advancements in PO (*i.e.*, single, double, and RP perovskite oxides) for SC applications. It also delves into the formation of composites and the increase in oxygen vacancy concentration to improve the electrochemical properties of PO. Despite the progress, several aspects still need to be addressed when designing future perovskite oxide electrode materials.

(1) Hydrothermal and solvothermal reactions are the primary techniques for synthesizing perovskite materials; however, their high energy requirements and costly reactants restrict large-scale synthesis. Perovskite materials with high purity and good uniformity can be synthesized using the most common synthesis techniques, such as sol–gel and solid-state methods, which use minimal energy. Therefore, it is essential to continue developing highly effective, environmentally friendly, and energy-efficient synthesis techniques.

(2) Perovskite materials are primarily determined by their oxygen vacancies, which can be created by doping, non-stoichiometric substitution, and other processes. Oxygen vacancies cause holes and redox pairs to form, which improves conductivity by promoting charge transfer. Moreover, more oxygen vacancies may promote pseudocapacitive qualities. Both A-site and B-site doping are practical methods for creating oxygen vacancies in perovskites, although B-site doping has been thoroughly and successfully investigated. In addition, excessive doping causes the crystal surface to segregate, and proper doping promotes the stability of the crystal lattice. Molecular and ionic doping can also enhance the electrochemical characteristics of perovskites. Future research must examine how co-doping A and B sites can create anionic vacancies as charge storage locations and achieve pseudo-capacitance in SCs.

(3) The morphology of the perovskite itself significantly influences the electrochemical properties of the composite. High charge mobility and quick electron transfer are made possible by the controlled scale, increased surface area, porous structure, and ion channels of nanostructures (flowers, nanoarrays, and nanorods). Carbon nanotubes (CNTs) and Activated Carbon (AC) with a large surface area were coupled with chalcocite materials to use perovskite as an electrode material for SCs with high energy and power densities and stability. The polymerization of polyazulene (PAz) on the surface of perovskite nanosheets enhances electron transfer by acting as a linker. This combination of organic and inorganic components shows both pseudo-capacitance and double-layer capacitance.

(4) The 3D bulk perovskite exhibits higher electronic conductivity than its 2D and 1D counterparts, primarily due to stronger electron-ion coupling and better orbital overlap. However, in terms of chemical and structural stability especially under environmental stressors like moisture or heat. The lower-dimensional 2D and 1D perovskites are generally more robust, owing to their layered or confined structures and often more hydrophobic organic components. Low-dimensional hybridized perovskites show promise as materials for charge storage while combining 2D and 3D materials preserves the former's stability and the latter's high efficiency.

(5) The electrochemical characteristics of the electrode material are also affected by the acidity or alkalinity of the electrolyte solution. In recent studies, perovskite bodies adsorb OH^−^ ions in the solution, releasing H^+^ ions and oxidizing them to O_2_, which, in turn, oxidizes other ions. Consequently, the alkaline electrolyte solution promotes the formation of oxygen vacancies and redox reactions. This process contributes to the high electrical conductivity observed in these materials.

(6) The electronic conductivity of RP perovskite oxides is expected to improve as their *n* value increases. However, most reports focus on RP perovskite oxides with *n* = 1. Consequently, RP perovskite oxides with higher *n* values are anticipated to be utilized as advanced SC electrode materials. Therefore, further efforts are needed to overcome the challenges presented by current research and to exploit novel perovskite materials for more promising applications.

## Abbreviations

SC: Supercapacitor
PO: Perovskite oxides
*C*_Sp_
: Specific capacitance

## Data availability

No new data were collected for this review.

## Author contributions

Mugil Neelakandan – writing – original draft. Preethi Dhandapani – writing – original draft. Senthilkumar Ramasamy – reviewing & editing. Seung Jun Lee & Ramesh Duraisamy – funding acquisition, resources, reviewing & editing. Subramania Angaiah – writing – review & editing, validation, supervision, resources, funding acquisition, conceptualization.

## Conflicts of interest

The authors declare no conflict of interest.
